# Prevalence and predictors for 72-h mortality after transfer to acute palliative care unit

**DOI:** 10.1007/s00520-022-07075-6

**Published:** 2022-05-02

**Authors:** Sebastian M. Christ, Minhtruong Huynh, Markus Schettle, Maiwand Ahmadsei, David Blum, Caroline Hertler, Annina Seiler

**Affiliations:** 1 Department of Radiation Oncology, University Hospital Zurich and University of Zurich, Zurich, Switzerland; 2 Competence Center Palliative Care, University Hospital Zurich and University of Zurich, Zurich, Switzerland; 3 Faculty of Medicine, University of Zurich, Zurich, Switzerland

**Keywords:** Palliative care, Short-term mortality, Survival prediction

## Abstract

**Purpose:**

Accurate prediction of survival is important to facilitate clinical decision-making and improve quality of care at the end of life. While it is well documented that survival prediction poses a challenge for treating physicians, the need for clinically valuable predictive factors has not been met. This study aims to quantify the prevalence of patient transfer 72 h before death onto the acute palliative care unit in a tertiary care center in Switzerland, and to identify factors predictive of 72-h mortality.

**Methods:**

All patients hospitalized between January and December 2020 on the acute palliative care unit of the Competence Center Palliative Care of the Department of Radiation Oncology at the University Hospital Zurich were assessed. Variables were retrieved from the electronic medical records. Univariable and multivariable logistic regressions were used to identify predictors of mortality.

**Results:**

A total of 398 patients were screened, of which 188 were assessed. Every fifth patient spent less than 72 h on the acute palliative care unit before death. In multivariable logistic regression analysis, predictors for 72-h mortality after transfer were no prior palliative care consult (p = 0.011), no advance care directive (p = 0.044), lower performance status (p = 0.035), lower self-care index (p = 0.003), and lower blood albumin level (p = 0.026).

**Conclusion:**

Late transfer to the acute palliative care unit is not uncommon, which can cause additional distress to patients and caretakers. Though clinically practical short-term survival predictors remain largely unidentified, early integration of palliative care should be practiced more regularly in patients with life-limiting illness.

**Supplementary Information:**

The online version contains supplementary material available at 10.1007/s00520-022-07075-6.

## Introduction and background

Approaching end-of-life (EoL) can be challenging for patients, relatives, nurses, and treating physicians alike [1]. Discussing when best to pivot from potentially life-prolonging therapy to palliative care may be complex, yet such topics should be on the agenda ideally earlier rather than later during the course of a life-limiting disease [2]. Often framed as advance care planning (ACP), these conversations may address goal of care, cardiopulmonary resuscitation, artificial nutrition, antibiotics, transfer to intensive care unit and acute palliative care units (APCUs), advance care directives, last will, last place of care and place of death [3, 4]. While it is impossible to plan for every detail arising within EoL, it is obvious that when all or many of these questions are left unaddressed until the very end, distress among patients, relatives, nurses and treating physicians may increase [5]. Yet even despite ACP and early palliative care integration, patients in large comprehensive cancer centers may be transferred to the APCUs days or even hours before passing away, thus limiting time for EoL preparation and often creating dissatisfactory situations for all stakeholders involved.

It is well documented that prediction of survival is a key component in the management of patients at the EoL. It is especially important for sensible decision-making, good resource allocation, and the improvement of quality of care [6, 7]. Prognostic awareness on the side of the patient has also been shown to positively influence individual treatment preferences [8]. Furthermore, knowing one’s prognosis is related to improved autonomy and to more satisfactory clinical outcomes [9, 10]. It is thus comprehensible and rational for patients and their relatives to frequently ask about prognosis, which presents treating physicians with a dilemma: They know of the importance of survival prediction, yet they are not good at it. There is ample evidence that physicians tend to systematically overstate survival in severely ill patients [11–13]. It has been shown that survival estimates of physicians are often correlated with actual survival, yet that physicians are better at forecasting the units of survival (days, weeks or months) rather than quantifying actual survival time [13, 14]. Results from large cohort studies suggest that physicians predict actual survival correctly in only about 20–25% of patients [13, 15].

Research efforts have therefore gone into identifying predictive factors and developing predictive tools to assist physicians in EoL decision-making. There have been attempts to interpret signs of impeding death [4], to better understand biological and physiological changes of the dying process [5], to retrospectively make sense of unexpected or sudden deaths [16, 17], and to develop models to forecast death when prescribing treatment [18]. Especially factors to predict short-term survival have been the focus of increasing clinical interest [7, 18–23]. However, prediction models for short-term survival have neither proven apt for clinical practice, nor been confirmed or validated in larger clinical trials. Here, the aim is to extend previous research efforts by quantifying the prevalence of 72-h mortality on the APCU in a large comprehensive cancer center in Switzerland to highlight the importance of better survival prediction and to assess the status of the current practice of multidisciplinary care integration. In a case–control design, we further aim at identifying predictive factors for death within 72 h from a range of variables including demographic, socio-economic, clinical and biological parameters.

## Materials and methods

### Study design and patient cohort

This retrospective single-center observational study was conceptualized as an unmatched case–control study. All patients who were hospitalized on the APCU of the Competence Center Palliative Care of the Department of Radiation Oncology at the University Hospital Zurich (USZ) between January and December 2020 were included in this study. Patients who died within 72 h of admission to the APCU (“outcome”) were identified as “cases”. Patients with a length of stay (LoS) of more than 60 days on the APCU were excluded, as they were taken not to be representative for the usual APCU patient clientele. From the remainder of patients, i.e., the group of patients who had a LoS between four and 59 days and thus did not exhibit the outcome (“death within 72 h of admission to palliative care”), the “control” group was selected. Simple random sampling using the = RANDBETWEEN() function in Microsoft® Excel® was employed as sampling methodology. A ratio of 1:1.4 of cases to controls, which lies within the commonly recommended range for case–control studies, was chosen by the research team [24, 25]. The authors decided against systematic case–control matching for the study cohort so as not to unnecessarily limit the number of controls and the analysis of possible risk factors. In addition, a small, by design underpowered, matched case–control sub-group analysis was undertaken to assess the persistence of identified effects. Sub-groups of 49 patients each (1:1 case to control ratio) were selected and matched on two variables, Eastern Cooperative Oncology Group Performance Status (ECOG-PS) and leading diagnosis.

### Data collection

A list of all patients hospitalized on the APCU in 2020 was available through the electronic medical records (EMR) KISIM™. Clinical parameters and biological markers of impending death were carefully selected based on clinical experience and a literature review of published studies and review articles [6, 14, 18, 26, 27]. For both the case and control group, all variables under study were manually extracted from the EMR. Demographic variables included an encoded unique patient identifier, date of birth, gender, and insurance status. Disease and treatment parameters included leading diagnosis, responsiveness at transfer, delirium status, oxygen requirement, ECOG-PS, self-care index (“SPI”), prior palliative care consultation, day of transfer to APCU, availability of advance care directive, C-reactive protein (CRP; mg/l) upon admission, albumin (mg/dl) upon admission, leukocyte count (G/L) upon admission, immature granulocyte count (G/L) upon admission, and thrombocyte count (G/L) upon admission. These values were routinely collected for all admitted patients unless ordered otherwise by the attending palliative care physician. Delirium was documented using the Delirium Observational Screening Scale (DOS). The ECOG-PS is a commonly used 5-point scale to assess the performance status of patients in oncological care [28]. The SPI comprises ten items, which represent a sub-set of the more comprehensive nursing tool “ergebnisorientierte PflegeAssessment AcuteCare^©^ (ePA-AC)”. Each item is scored on a 4-point scale, resulting in scores ranging from ten (“complete dependence”) to 40 (“complete independence”) points. Additional variables like source department, total inpatient LoS in days, LoS in days on the APCU, date of admission to the APCU, date of discharge from the APCU, and place of death were retrieved from the accounting department. Ambiguous parameters were reviewed by at least two researchers to guarantee consistency of data entry across the whole cohort. The spreadsheet program Microsoft® Excel® (Version 16.0) was used to compile the data. Upon extraction of the data, all data were encoded. This study, which is part of a research project series on quality-of-life in palliative care patients, was approved by the Swiss Cantonal Ethics Committee before initiation of the project (BASEC ID #2019–02,488).

### Statistical analysis

Appropriate descriptive summary statistics were computed for all demographic, socioeconomic, clinical and biological variables. The normality assumption was assessed graphically and computationally for all variables under study. To assess statistically significant differences between the case and the control group, the parametric student t-test was used for normally distributed variables; for non-normally distributed variables, the nonparametric Mann–Whitney U test and the Wilcoxon rank-sum test were employed. Statistical significance was set at p < 0.05, as common in the medical literature. Univariable logistic regression analysis was used to assess potential predictors of death within 72 h after admission to the APCU. Variables for which a significant difference between the case and control group were identified, were included into the logistic regression analysis. All continuous or multi-categorial variables were categorized based on common cut-offs or clinically employed thresholds. Multivariable logistic regression analysis was conducted using the backward method. For the matched sub-group analysis, the same statistical methodology was followed. The statistical software package STATA® (Version 16.1.) was used for all statistical calculations.

## Results

Between January and December 2020, a total of 398 patients were hospitalized on the APCU of the USZ. Seventy-eight patients (20%) died within 72 h of transfer to the APCU. The control group consisted of 110 (25%) patients. This brings the total of assessed patients to 188. Owing to the design of this study, the median LoS on the APCU for the case group was 2 days (interquartile range (IQR), 2–3 days); the LoS for the control group was 9 days (IQR, 6–15 days).

### Basic patient characteristics

The median age of the whole patient cohort was 70 years (IQR, 61–79 years), and 57% of patients were male. There was no significant difference in age and gender between case and control groups. The proportion of patients with malignant disease was significantly higher in the control than in the case group, with an oncological diagnosis present in 84% and 56% of patients, respectively (p < 0.001). There was also a significant difference in the ECOG-PS between groups: In the case group, more than 80% of patients had ECOG-PS 4, while in the control group only 41% had ECOG-PS 4 (p < 0.001). In both the case and the control group, the proportion of patients with general public insurance was above 80%, yet while there was only one patient privately insured in the case group, there were twelve patients with private insurance in the control group (p = 0.004). For a summary of basic patient characteristics by group, consult Table 1.

**Table 1:** Summary of basic patient characteristics by group

Variable	Case	Control	Total	*p-value*
Age; median (IQR)	73 (62–81)	70 (61–78)	70 (61–79)	0.295
Gender; n (%)				0.906
Male	44 (56)	63 (57)	107 (57)	
Female	34 (44)	47 (43)	81 (43)	
Leading diagnosis; n (%)				< 0.001
Malignancy	44 (56)	92 (84)	136 (72)	
Non-oncological disease^1^	34 (44)	18 (16)	52 (28)	
ECOG-PS; n (%)				< 0.001
0–1	0 (0)	7 (6)	7 (4)	
2–3	15 (19)	58 (53)	73 (39)	
4	63 (81)	45 (41)	108 (57)	
Insurance status; n (%)				0.004
General public	65 (83)	92 (84)	157 (84)	
Half-private	12 (15)	6 (5)	18 (10)	
Private	1 (1)	12 (11)	13 (7)	

IQR = Inter-quartile range; ECOG-PS = Eastern Cooperative Oncology Group Performance Status

^1^ Includes all non-malignant disease such as chronic heart, kidney and endocrinological disease as well as various neurological conditions

### Service-related variables

Patients were most commonly transferred from the Hemato-Oncology (28%) and the Emergency department (10%), with the remaining 117 patients (62%) coming from other clinical departments. The large majority (89%) of transfers occurred on weekdays. While there was no significant difference in source department and day of transfer between the case and control group, there were statistically significant differences in prior palliative care consult and availability of advance care directives: While 46% of cases had a prior palliative care consult, the proportion in the control group was significantly higher with 62% (p = 0.033). In the case group, only 29 (37%) patients had completed advance care directives, whereas the control group (N = 63; 57%) was more than twice as likely to do so (p = 0.020). For a summary of service-related variables, see Table 2.

**Table 2:** Summary of service-related variables by group

Variable	Case	Control	Total	*p-value*
Source department; n (%)				0.074
Hematology-Oncology	21 (27)	32 (29)	53 (28)	
Emergency department	12 (15)	6 (5)	18 (10)	
Other^1^	45 (58)	72 (65)	117 (62)	
Day of transfer; n (%)				0.738
Weekday	70 (90)	97 (88)	167 (89)	
Weekend	8 (10)	13 (12)	21 (11)	
Prior palliative care consult; n (%)				0.033
Yes	36 (46)	68 (62)	104 (55)	
No	42 (54)	42 (38)	84 (45)	
Advance care directive; n (%)				0.020
Yes	29 (37)	63 (57)	92 (49)	
No	49 (63)	47 (43)	96 (51)	

IQR = Inter-quartile range

^1^ Includes the rest of internal medicine sub-specialties and all surgical disciplines, among others

### Clinical and biological variables

The case and control patient cohorts differed with respect to various clinical and biological variables under study. The ability for self-care, captured by the SPI, was significantly different between the two groups: In the case group, 67 patients (86%) had a SPI between 10 and 19 points, with only 14% (N = 11) of patients with a SPI larger than 20 points. In the control group, 45 patients (41%) had a SPI between 10 and 19 points, and 65 patients (59%) had a SPI larger than 20 points (p < 0.001). When it comes to responsiveness at transfer, patients in the control group (N = 85; 77%) were significantly more responsive than patients in the case group (N = 25; 23%) (p < 0.001). With respect to a delirious state and oxygen requirement upon admission, there was no significant difference between case and control groups (p = 0.731). In both groups, less than 20% of patients were delirious, and 35% and 45% of patients in the case and the control group, respectively, were given supplemental oxygen.

Biological markers were available for sub-groups of patients only. CRP upon admission was available for 151 patients. The Median CRP was elevated at 100 mg/l (27–189 mg/l) and 95 mg/l (33–168 mg/l) in the case and control group, respectively, with the slight difference not being statistically significant (p = 0.872). Albumin levels were available for 110 patients, and the difference of 26 mg/dl (21–31 mg/dl) in the case group and 29 mg/dl (26–34 mg/dl) in the control group was significantly different (p = 0.016). Leucocyte, immature granulocyte and thrombocyte counts upon admission were available only for 152, 123 and 151 patients, respectively, and the detected differences did not prove to be statistically different (p = 0.515; p = 0.771; p = 0.450). For a summary of clinical and biological variables, compare Table 3.

**Table 3:** Summary of clinical and biological variables by group

Variable	Total	Case	Control	*p-value*
SPI; n (%)				** < **0.001
40–30	36 (19)	4 (5)	32 (29)	
29–20	40 (21)	7 (9)	33 (30)	
19–10	112 (60)	67 (86)	45 (41)	
Responsiveness; n (%)				** < **0.001
Yes	122 (65)	37 (47)	85 (77)	
No	66 (35)	41 (53)	25 (23)	
Delirium; n (%)				0.731
Yes	31 (16)	12 (15)	19 (17)	
No	157 (84)	66 (85)	91 (83)	
Oxygen requirement; n (%)				0.255
Yes	76 (40)	27 (35)	49 (45)	
No	112 (60)	51 (65)	61 (55)	
CRP; median (IQR); *[n]*	100 (29–180) *[151]*	100 (27–189) *[68]*	95 (33–168) *[83]*	0.872
Albumin; median (IQR); *[n]*	28 (24–33) *[110]*	26 (21–31) *[52]*	29 (26–34) *[58]*	0.016
Leucocytes; median (IQR); *[n]*	10 (7–16) *[152]*	12 (8–19) *[68]*	9 (7–14) *[84]*	0.515
Immature granulocytes; median (IQR); *[n]*	0.15 (0.07–0.41 *[123]*	0.19 (0.08–0.50) *[59]*	0.14 (0.06–0.29) *[64]*	0.771
Thrombocytes; median (IQR); *[n]*	212 (123–278) *[151]*	207 (90–274) *[67]*	218 (153–280) *[84]*	0.450

CRP = C-reactive protein; IQR = Inter-quartile range; SPI = Self-care index

^1^ Includes all non-malignant disease such as chronic heart, kidney and endocrinological disease as well as various neurological conditions

### Univariable logistic regression analysis

In univariable logistic analysis, seven out of the eight examined variables were significant. A prior palliative care consult had an odds ratio (OR) of 0.529 (95% confidence interval (CI), 0.294–0.953) for 72-h mortality after transfer to the APCU (p = 0.034). The availability of an advance care directive at the time of admission was an OR of 0.438 (95% CI, 0.243–0.788) with an associated significance of p = 0.006. Insurance status, categorized as general public versus (vs.) private insurance, had an OR of 1.022 (95% CI, 0.468–2.232) and was not significant (p = 0.956). In univariable analysis, a non-malignant leading diagnosis, an ECOG-PS of 4 or higher, a SPI of 19 points and lower, and no responsiveness upon admission were all significantly associated with 72-h morality upon transfer to acute palliative care, though actual effect sizes vary greatly (p = 0.000). The OR for no malignant vs. malignant leading diagnosis was 3.949 (95% CI, 2.011–7.756), the OR for ECOG–PS 4–5 vs. 0–3 was 6.067 (3.075–11.967), the OR for SPI 20–40 vs. 10–19 points was 0.114 (95% CI, 0.054–0.238), and the OR for responsiveness vs. no responsiveness was 0.265 (95% CI, 0.141–0.498). A higher (> 26 mg/dl) vs. a lower (< 26 mg/dl) albumin level at the time of admission in 110 of 188 patients was significantly associated with 72-h mortality (p = 0.016), with an OR of 0.356 (0.153–0.824) in univariable logistic regression.

### Multivariable logistic regression analysis

In multivariable logistic regression analysis, a significant effect persisted for five variables. Predictors for 72-h mortality after transfer to palliative care were no prior palliative care consult (p = 0.011) with an OR of 0.162 (0.039–0.657), no advance care directive (p = 0.044) with an OR of 0.217 (0.049–0.957), a numerically higher ECOG-PS (p = 0.035) with an OR of 3.661 (1.097–12.214), a lower SPI (p = 0.003) with an OR of 0.167 (0.051–0.547), and a lower albumin level (p = 0.026) with an OR of 0.298 (0.102–0.866). Leading diagnosis and responsiveness at the time of admission were not significant in multivariable logistic regression analysis. For an overview of regression results, see Table 4.

**Table 4:** Univariable and multivariable predictor analysis

Variable	Univariable analysis		Multivariable analysis	
	OR (95% CI)	*p-value*	OR (95% CI)	*p-value*
Prior palliative care consult	0.529 (0.294–0.953)	**0.034**	0.162 (0.039–0.657)	**0.011**
Yes vs. No				
Advance care directive	0.438 (0.243–0.788)	**0.006**	0.217 (0.049–0.957)	**0.044**
Yes vs. No				
Insurance status	1.022 (0.468–2.232)	0.956	N/A	N/A
General public vs. Private				
Leading diagnosis	3.949 (2.011–7.756)	** < 0.001**	5.081 (0.805–32.078)	0.084
No malignancy vs. malignancy				
ECOG-PS	6.067 (3.075–11.967)	** < 0.001**	3.661 (1.097–12.214)	**0.035**
4 vs. 0–3				
SPI	8.798 (4.188–18.482)	** < 0.001**	4.831 (1.595–14.636)	**0.005**
10–19 vs. 20–40				
Responsiveness	0.265 (0.141–0.498)	** < 0.001**	1.811 (0.496–6.605)	0.368
Yes vs. No				
Albumin	0.356 (0.153–0.824)	**0.016**	0.298 (0.102–0.866)	**0.026**
> 26 mg/dl vs. < 26 mg/dl				

CI = Confidence interval; ECOG-PS = Eastern Cooperative Oncology Group Performance Status; OR = Odds ratio; SPI = Self-care index

### Matched case–control sub-group analysis

By design, the case and control sub-groups were similar in terms of basic patient characteristics, service-related variables, as well as biological and clinical factors. Only the SPI differed significantly between both sub-groups (p = 0.037). On univariable logistic regression analysis, SPI (p = 0.036) and albumin levels (p = 0.044) were the only variables significantly associated with the outcome. On multivariable logistic regression analysis, SPI was no longer, yet prior palliative care consult (p = 0.049) was significantly associated with the outcome. The albumin level effect (p = 0.026) persisted on multivariable analysis. ECOG-PS and leading diagnosis could not be assessed due to the nature of the study design, as they were used to match the two sub-groups. For a summary of the sub-group analysis, consult the Supplementary Tables (1–4).

## Discussion

In this retrospective study, the prevalence of and predictors for 72-h mortality after transfer to the APCU at a large comprehensive cancer center in Switzerland are assessed. Our data show that 20% of transferred patients died within 72 h of arrival on the APCU in 2020, and in multivariable regression analysis, five variables proved to be significantly associated with 72-h mortality, suggesting they have predictive value.

Reasons for patients being transferred within the last days or hours of their life may have multiple causes, ranging from erroneous survival prediction by physicians, lack of prognostic awareness by patients and their relatives, and structural factors at work in large cancer centers. It is striking to see that even after prior consultation via the palliative care team in many cases, every fifth patient died within 72 h after arrival on the APCU. Other studies have reported on similar experiences: *Bruera *et al*.* (2015) found that 10% of patients had died unexpectedly shortly after transfer to the APCU according to treating physicians at the M.D. Anderson Cancer Center in Houston, Texas, USA in 2010 [16]. *Goncalves *et al*.* (2003) reported a rate of 9% of patients which died within 48 h of transfer to the APCU at the Portuguese Institute of Oncology in Porto, Portugal between 1995 and 1998 [29]. While death within 72 h of transfer of severely injured patients from the emergency department or the intensive care unit may be rationalized and arguably represent an important service in a tertiary university hospital, it is debatable whether patients from other wards, who stay on the APCU for less than 72 h, can profit from the wide range of services modern integrative palliative care offers.

In multivariable analysis, a numerically lower ECOG-PS, a numerically higher SPI, a higher albumin level, an advance care directive, and a prior palliative care consult were all significantly protective against 72-h mortality after transfer to the APCU. With respect to performance status, our findings are in line with other studies. In a systemic review conducted by *Vigano *et al*.* (2000) already several years ago, the predictive power of a patient’s performance status was confirmed. At the time, the authors also pointed out the heterogeneity of the use of the performance status—ECOG-PS vs. Karnofsky Performance Score (KPS)—and the random cut-off scores chosen to partition patient sub-groups in different publications [30]. The performance status also has significant predictive value in newer studies, while the variation in use seems to persist to this day [5, 6, 19]. Despite these nuances discussed in the literature, the performance status is accepted as a good predictive marker for short-term mortality.

Less commonly employed and even less standardized than the performance score is the SPI, an example of a self-care index, which is compiled by nursing staff. The lesser patients are able to care for themselves and look after their basic needs, the worse their general state of health. With nursing staff spending a lot of time around patients, it is not surprising that the SPI scoring has predictive value. In a study by *Hui *et al*.* (2015), eight physical signs, which had a high specificity and a high likelihood ratio for death within the last three days of life, were identified and repeatedly scored by closely involved caretakers. These factors included decreased response to verbal and visual stimuli, inability to close eyelids, non-reactive pupils, drooping of the nasolabial fold, hyperextension of the neck, grunting of vocal cords, and upper gastrointestinal bleeding [31]. Like the SPI, scoring many of these mostly observable symptoms and bodily signs rely on subjective ratings, which may be impacted by the time of assessment and the experience and knowledge of the observer.

Rather than subjective measures, objective measures are what is needed in order to consistently improve short-term survival prediction. There are few studies that have evaluated and successfully identified objective predictive factors for short-term survival in patients with advanced diseases. Several years ago, *Bruera *et al*.* (1992) determined three factors as indicators for poor prognosis in a palliative cohort of patients: dysphagia, cognitive failure as measured by the Mini Mental State Examination (MMSE), and weight loss greater than 10 kg [32]. *Vigano *et al*.* (2000) pointed out more than twenty years ago that many studies postulate that symptoms associated with the “terminal cancer syndrome theory” or the “anorexia-cachexia syndrome” (ACS) such as dysphagia, nausea, emesis, anorexia, or cachexia are important for survival prediction [30]. *Chen *et al*.* (2015), in a more recent publication, identified six objective predictors for 7-day mortality: heart rate, leukocyte count, platelet count, serum creatinine, serum potassium, and a history of chemotherapy [19]. Other studies found biological factors such as leukocytosis, lymphocytopenia, albumin levels, serum lactate dehydrogenase levels, and CRP levels to carry predictive value [6, 30]. While MMSE scores and symptoms associated with ACS may carry predictive value for survival prediction in general, they do not qualify as predictors for 72-h mortality, as they tend to decisively change over the course of months, weeks or days, yet not during the last hours of life, where they are usually not recorded anymore anyways. Our study validated lower albumin levels to carry predictive value for short-term survival, yet other biological markers like CRP level, leucocyte count, immature granulocyte count, and thrombocyte count did not have predictive value in this patient cohort. One challenge in comparing predictors across these patient series is reconciling the variation in study designs, examined predictor variables, used assessment tools and frequencies as well as the clinical settings. Also, as other authors have rightly pointed out, when wanting to compile short-term survival prediction tools, it should be kept in mind that not everything which carries predictive value and is interesting to measure invasively, should in fact be measured—it may be inappropriate to do so, as it may results in additional discomfort in the patient [4].

Two predictor variables, which stand out among all others in this study, are the service-related variables prior palliative care consultation and advance care directives. In case a patient had previously filled an advance care directive or been consulted by a specialist of the palliative care service team before being transferred to the APCU, had a reduced 72-h mortality after transfer to the APCU. This finding indicates that ACP and planning for EoL as well as earlier integration of palliative care have the potential to help patients and relatives reserve time and put the focus on the subjective needs during the EoL period. While a transfer to APCU within the last 72 h may not be too late for every patient, chances that both patient and relatives profit are higher if the transfer occurs sooner.

The unmatched design of this case–control study allowed the authors to assess every possible association of variables, incl. those, which differed significantly between the case and control groups, such as ECOG-PS and leading diagnosis. Both were significantly associated with the study outcome, i.e., death within 72 h after transfer to palliative care, on univariable analysis, for ECOG-PS the effect carried over into multivariable analysis. The effect size for the leading diagnosis variable increased from univariable to multivariable analysis, highlighting that it might indeed carry predictive value, yet confounding preventing the detection of a significant signal. When controlling for their potential confounding via an underpowered, matched-control sub-group analysis, the identified associations between the outcome and prior palliative care consult, SPI and albumin level persisted on univariable analysis, multivariable analysis or both. The effect of ECOG-PS and leading diagnosis could not be evaluated in sub-group analysis, as they were used to match sub-groups. They have been shown to be predictive in other studies [5, 6, 19].

It is a strength of this unmatched case–control study to have assessed a large number of potential predictors for the study outcome variable. Shortcomings of this study mainly stem from its retrospective nature: The small sample size and the retrospective analysis limit the generalizability of the results of the study. This applies especially for the underpowered sub-group analysis. In addition, for some analyses, the sample size in the logistic regression models was further reduced, because not all values were present for all patients, especially with respect to biological markers. While the sample size was still adequate to ensure power, some CIs ended up being quite wide, and not all identified effects were of an impactful size.

In conclusion, transfer to the APCU in our large comprehensive cancer center in Switzerland within 72 h of death is not uncommon. Every fifth patient spends less than three days on the integrative palliative care service, creating additional distress to patients, relatives and caretakers alike. In the absence of clinically practical short-term survival predictors, physicians should even more so aim for early palliative care integration and encourage the completion of advance care directives. These two service-related variables should also feature in future prediction tools in order to improve participatory decision-making and quality-of-care in the clinical EoL setting.

## Supplementary Information

Below is the link to the electronic supplementary material.

Supplementary file1 (DOCX 21 KB)

## Data Availability

Collected patient data are private and not available for publication.

## Abbreviations

ACP: Advance care planning
ACS: Anorexia-cachexia syndrome
APCU: Acute Palliative Care Unit
BASEC: Business Administration System for Ethics Committees
CI: Confidence interval
CRP: C-reactive protein
DOS: Delirium Observational Screening Scale
ECOG-PS: Eastern Cooperative Oncology Group Performance Status
EoL: End-of-life
EMR: Electronic medical records
IQR: Interquartile range
KPS: Karnofsky Performance Score
LoS: Length of stay
MMSE: Mini Mental State Examination
OR: Odds ratio
SPI: Self-care index
USZ: University Hospital Zurich
VS: Versus

## Code availability

Not applicable for this publication.
