# Supplementary material for: Prevalence and predictors for 72-h mortality after transfer to acute palliative care unit
Source: Support Care Cancer. 2022 May 2;30(8):6623–31. doi: 10.1007/s00520-022-07075-6 (PMC9213309; doi:10.1007/s00520-022-07075-6)
Supplement: Supplementary file 1 — Supplementary file1 (DOCX 21 KB) [file 520_2022_7075_MOESM1_ESM.docx]

**Supplements**

**Supplementary table 1.** Summary of basic patient characteristics for matched sub-group

| Variable | Case  (n=49) | Control  (n=49) | Total  (n=98) | p-value |
| --- | --- | --- | --- | --- |
| Age; median (IQR) | 73 (59–82) | 74 (62–84) | 73 (61–83) | 0.812 |
| Gender; n (%) |  |  |  |  |
| - Male | 21 (43) | 19 (39) | 40 (41) | 0.683 |
| - Female | 28 (57) | 30 (61) | 58 (59) |  |
| Leading diagnosis; n (%) |  |  |  | 0.308 |
| - Malignancy | 26 (53) | 31 (63) | 57 (58) |  |
| - Non-oncological disease^1^ | 23 (47) | 18 (37) | 41 (42) |  |
| ECOG-PS; n (%) |  |  |  | 0.976 |
| - 0-1 | 0 (0) | 0 (0) | 0 (0) |  |
| - 2-3 | 4 (8) | 4 (8) | 8 (8) |  |
| - 4 | 45 (92) | 45 (92) | 90 (92) |  |
| Insurance status; n (%) |  |  |  | 0.071 |
| - General public | 40 (82) | 41 (84) | 81 (83) |  |
| - Half-private | 9 (18) | 2 (4) | 11 (11) |  |
| - Private | 0 (0) | 6 (6) | 6 (6) |  |

Abbreviations: IQR = Inter-quartile range; ECOG-PS = Eastern Cooperative Oncology Group Performance Status.

^1^ Includes all non-malignant disease such as chronic heart, kidney and endocrinological disease as well as various neurological conditions.

**Supplementary table 2.** Summary of service-related variables for matched sub-group

| Variable | Case  (n=49) | Control  (n=49) | Total  (n=98) | p-value |
| --- | --- | --- | --- | --- |
| Source department; n (%) |  |  |  | 0.471 |
| - Hematology-Oncology | 11 (22) | 10 (20) | 21 (21) |  |
| - Emergency department | 8 (16) | 5 (10) | 13 (13) |  |
| - Other^1^ | 30 (61) | 34 (69) | 64 (65) |  |
| Day of transfer; n (%) |  |  |  | 0.300 |
| - Weekday | 46 (94) | 43 (88) | 89 (91) |  |
| - Weekend | 3 (6) | 6 (12) | 9 (9) |  |
| Prior palliative care consult; n (%) |  |  |  | 0.068 |
| - Yes | 23 (47) | 32 (65) | 55 (56) |  |
| - No | 26 (53) | 17 (35) | 43 (44) |  |
| Advance care directive; n (%) |  |  |  | 0.311 |
| - Yes | 19 (39) | 24 (49) | 43 (44) |  |
| - No | 30 (61) | 25 (51) | 55 (56) |  |

Abbreviations: IQR = Inter-quartile range.

^1^ Includes the rest of internal medicine sub-specialties and all surgical disciplines, among others.

**Supplementary table 3.** Summary of clinical and biological variables for matched sub-group

| Variable | Case | Control | Total | p-value |
| --- | --- | --- | --- | --- |
| SPI; n (%) |  |  |  | **0.0369** |
| - 40-30 | 3 (6) | 6 (12) | 9 (9) |  |
| - 29-20 | 4 (8) | 10 (21) | 14 (14) |  |
| - 19-10 | 42 (86) | 33 (67) | 75 (77) |  |
| Responsiveness; n (%) |  |  |  | 0.315 |
| - Yes | 22 (45) | 27 (55) | 49 (50) |  |
| - No | 27 (55) | 22 (45) | 49 (50) |  |
| Delirium; n (%) |  |  |  | 0.139 |
| - Yes | 4 (8) | 9 (18) | 13 (13) |  |
| - No | 45 (92) | 40 (82) | 85 (87) |  |
| Oxygen requirement; n (%) |  |  |  | 0.278 |
| - Yes | 13 (27) | 18 (37) | 31 (32) |  |
| - No | 36 (73) | 31 (63) | 67 (68) |  |
| CRP;  median (IQR); [n] | 100 (20–199)  [n=43] | 84 (27–144)  [n=37] | 89 (26–169)  [n=80] | **0.043** |
| Albumin;  median (IQR); [n] | 26 (21–32)  [n=31] | 30 (26–36)  [n=26] | 28 (24–33)  [n=57] | 0.059 |
| Leucocytes;  median (IQR); [n] | 12 (8–19)  [n=43] | 10 (8–14)  [n=37] | 11 (8–17)  [n=80] | 0.712 |
| Immature granulocytes;  median (IQR); [n] | 0.22 (0.09–0.49) [n=37] | 0.11 (0.06–0.20) [n=27] | 0.15 (0.08–0.42) [n=64] | **0.049** |
| Thrombocytes;  median (IQR); [n] | 207 (94–287)  [n=43] | 206 (128–270)  [n=37] | 207 (99–294)  [n=80] | 0.607 |

Abbreviations: CRP = C-reactive protein; IQR = Inter-quartile range; SPI = Self-care index.

^1^ Includes all non-malignant disease such as chronic heart, kidney and endocrinological disease as well as various neurological conditions.

**Supplementary table 4.** Univariable and multivariable predictor analysis for matched sub-group

| Variable | Univariable analysis | | Multivariable analysis | |
| --- | --- | --- | --- | --- |
|  | OR  (95% CI) | p-value | OR  (95% CI) | p-value |
| Prior palliative care consult | 0.470  [0.208–1.059] | 0.069 | 0.284  [0.081–0.995] | **0.049** |
| - Yes vs. No |  |  |  |  |
| Advance care directive | 0.660  [0.296–1.472] | 0.310 | 0.916  [0.272–3.083] | 0.888 |
| - Yes vs. No |  |  |  |  |
| Insurance status | 1.153  [0.405–3.286] | 0.790 | 1.502  [0.288–7.819] | 0.629 |
| - General public vs. Private |  |  |  |  |
| SPI | 2.909  [1.072–7.895] | **0.030** | 2.791  [0.621–12.539] | 0.082 |
| - 10-19 vs. 20-40 |  |  |  |  |
| Responsiveness | 0.664  [0.299–1.472] | 0.476 | 1.609  [0.398–6.502] | 0.504 |
| - Yes vs. No |  |  |  |  |
| Albumin | 0.289  [0.087–0.965] | **0.044** | 0.218  [0.569–0.836] | **0.026** |
| - >26mg/dl vs. <26mg/dl |  |  |  |  |

Abbreviations: CI = Confidence interval; OR = Odds ratio; SPI = Self-care index.
